# The Company of Biologists: celebrating 100 years

**DOI:** 10.1242/dmm.052228

**Published:** 2025-01-06

**Authors:** Sarah J. Bray, Stephen J. Royle, Holly A. Shiels, Daniel St Johnston

**Affiliations:** ^1^ Department of Physiology Development and Neuroscience, University of Cambridge, Downing Street, Cambridge CB2 3DY, UK; ^2^ Warwick Medical School, University of Warwick, Gibbet Hill Road, Coventry CV4 7AL, UK; ^3^ Faculty of Biology, Medicine, and Health, Core Technology Facility, 46 Grafton Street, University of Manchester, Manchester M13 9NT, UK; ^4^ The Gurdon Institute, University of Cambridge, Tennis Court Rd, Cambridge CB2 1QN, UK

## Abstract

**Summary:** As we begin celebrating our 100-year anniversary, we reflect on our extraordinary story so far, consider the Company's current support for our communities and prepare for some inspiring activities.

**Figure:**
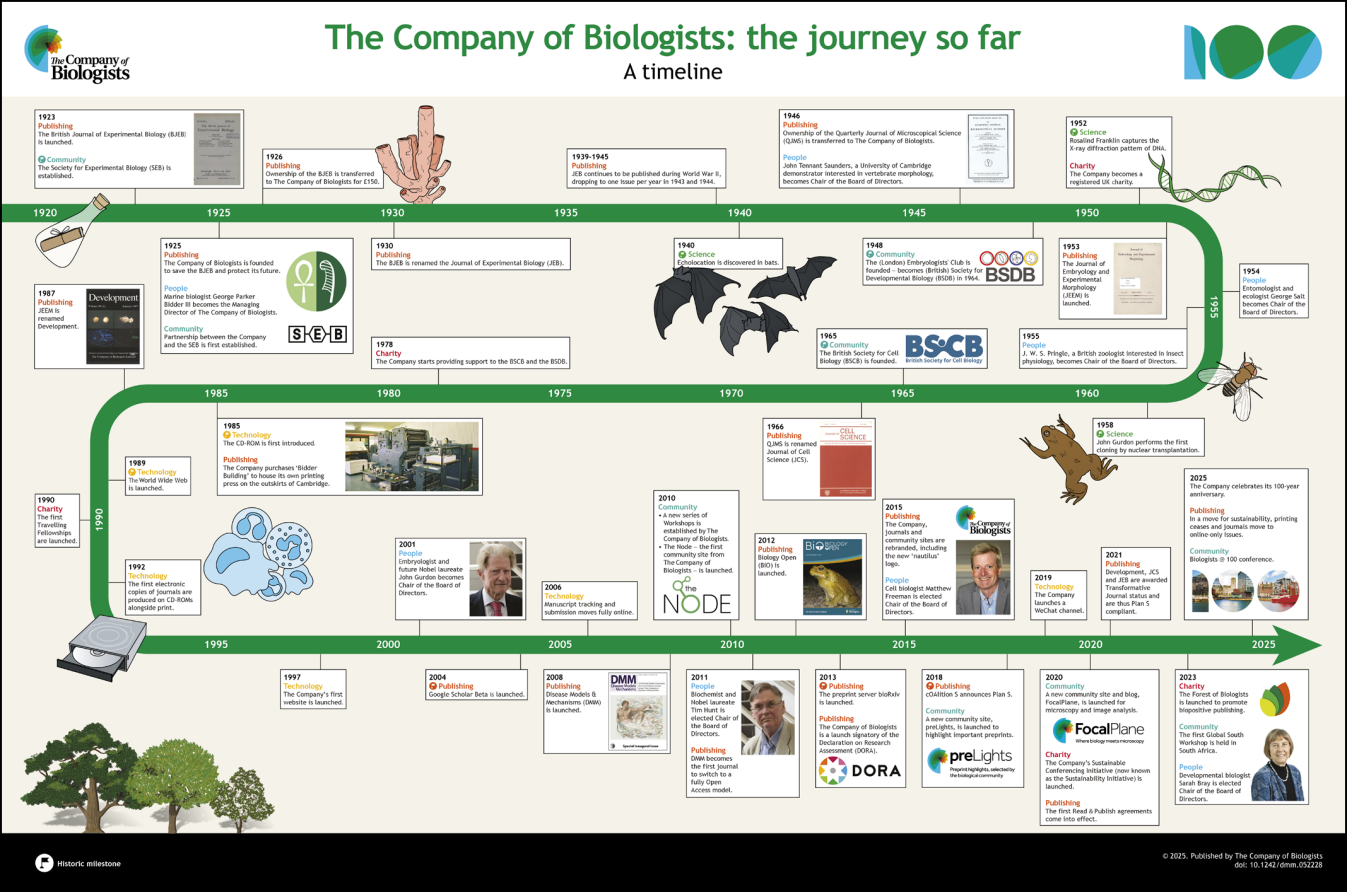
See supplementary information for a high-resolution version of the poster.

2025 marks the 100-year anniversary of the founding of The Company of Biologists and what an extraordinary voyage it has been. Founded by biologists to promote science, our core ethos remains to support the community by publishing top-notch science and by providing grants to sustain scientific societies and community activities. As part of our celebrations, we are publishing articles about our past, present and future in each of our five journals: Development, Journal of Cell Science, Journal of Experimental Biology, Disease Models & Mechanisms and Biology Open. These articles will be complemented by posts on our community sites (the Node, preLights and FocalPlane), as well as stories on our website. To see all our celebratory activities, please visit our anniversary page: https://www.biologists.com/100-years/. In this initial, cross-title Editorial, four of our current Directors look back through events in our 100-year history with an accompanying poster timeline that details the Company's journey so far. We also summarise some of our present activities and look forward to our future challenges and goals.

## From humble beginnings: a brief history of The Company of Biologists

The Company of Biologists' story begins, in fact, 102 years ago with the launch of a then-new journal – The British Journal of Experimental Biology (BJEB) – by zoologists Lancelot Hogben, Francis (Frank) Crew and Julian Huxley. BJEB quickly fell into financial difficulty due to the launch of a competitor journal, Biological Proceedings, led by fellow zoologist James Gray. Crew encouraged marine biologist George Parker Bidder III (Box 1) to convince Gray to merge the publications and save the BJEB. Bidder agreed on the condition that the journal should be owned by a company specifically founded for the purpose. In an early example of crowdfunding, Bidder recruited 38 friends and colleagues to buy £5 shares in the new company, and thus The Company of Biologists was formed (Skaer, 2001; Erlingsson, 2013; Knight, 2023).

**Box 1. George Parker Bidder III**

Born in London in 1863, George Parker Bidder III was a British marine biologist primarily interested in researching hydraulics in sponges. As a founding member of the Society for Experimental Biology (SEB) and later President of the Marine Biological Association in Plymouth, Bidder was a prominent figure in the field of zoology, marine biology and ecology. It was such positions that first led the Editors of The British Journal of Experimental Biology to seek his help in saving their journal, which he did by founding The Company of Biologists in 1925. He acted as Managing Director and Secretary of the Company until 1928, remaining on the Board until his resignation in 1942. Bidder died aged 90 in 1953 in Cambridge, UK. The Company commemorated his contribution with a short series of ‘George Bidder Lectures’ in the 1970s, organised with the SEB and published in Journal of Experimental Biology (Wigglesworth, 1971). Bidder entered public news once again when, in April 2015, the oldest message in a bottle was discovered – one of 1000 released by Bidder between 1904 and 1906 while studying North Sea currents. We are using this theme during our 100-year anniversary to gather feedback and stories from our communities. To find out how to send your own ‘message in a bottle’, visit our anniversary page: https://www.biologists.com/100-years/. Today, The Company of Biologists acknowledges Bidder’s contribution by naming our offices ‘Bidder Building’. He also features as part of our ‘100 extraordinary biologists’ 2025 campaign, recognising 100 researchers with extraordinary links to The Company of Biologists; follow #100biologists on social media or see the collection as it grows on our anniversary page.
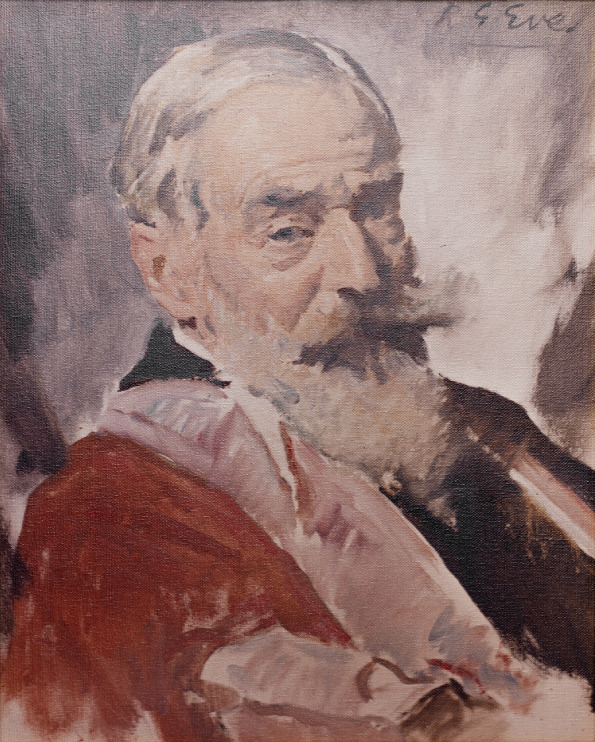

**Fig. 1.**
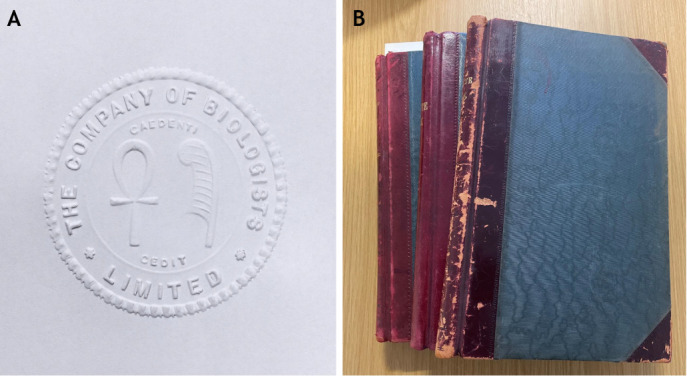
**Some relics from The Company of Biologists.** (A) The Company seal, featuring two Egyptian symbols – the ankh (representing life) and the feather (representing the goddess Maat as a symbol for truth, balance and order) – is kept under lock and key. The key, originally held by George Parker Bidder III, was lost by subsequent secretaries on more than one occasion. (B) Some of the handwritten minute books recording meetings held by the Board, and a source of much of the information in the history portion of this Editorial.

The first recorded meeting was held on 25 October 1925 at Bidder's home, Cavendish Corner, in Cambridge, UK, where Bidder produced the Company seal (Fig. 1A), the first minute book (Fig. 1B), a register of members and certificates of shares. The following year, the transfer of the BJEB from Crew, Huxley and Hogben to The Company of Biologists was agreed – the Company owned its first journal. For more on what lay ahead of the future of the BJEB, now known as Journal of Experimental Biology (JEB), see Knight (2023), and for the full collection of articles celebrating JEB's centenary, please visit https://journals.biologists.com/jeb/collection/9016/JEB-100-Years-of-Discovery.

The first year of trading saw a ‘satisfactory’ profit of over £50 (equivalent to around £2500 today). However, difficulty struck the following year with a reported loss of over £110. The turbulent early years of the Company's finances were loosely stabilised by Government Grants via the Royal Society until such a time that the Company could no longer ‘plead poverty’ and thus was no longer eligible. There was also some confusion regarding eligibility for Royal Society funding because of the Company's ‘indefinite’ relationship with the Society for Experimental Biology (SEB). Bidder and other board members were founding members of the SEB and the two entities often overlapped. Early on, the SEB provided additional funds to support the Company in exchange for services, such as journal pages for printing output from their symposia. Today, the Company supports the SEB with an annual charitable grant. In the late 1970s, the Company also forged relationships with the British Society for Cell Biology (BSCB) and the British Society for Developmental Biology (BSDB), which continue today. More on how we support biological societies will follow in articles published throughout this year.

Bidder served as the Company Secretary and Managing Director from 1925 until 1928, during which period the Board would continue to meet regularly at Cavendish Corner, and then at University Colleges in Cambridge and London as the Secretary's pen changed hands. He continued to be intimately involved in the Company, until his resignation in 1942, often taking handwritten minutes of the meetings.

However, Bidder's departure from the Board did not mark the end of his involvement with the Company. Somehow, Bidder had come to own the Quarterly Journal of Microscopical Science (QJMS), which he gifted to The Company of Biologists in 1946, and it was renamed Journal of Cell Science (JCS) 20 years later. The journal's long history will be discussed in more detail in an article published in JCS next month.

Aside from acquiring established journals, the Company was approached several times to publish new journals, none of which made it to print. However, in 1952, the same year that the Company transitioned to becoming a registered charity, the Board agreed to a proposal for a new journal: the Journal of Embryology and Experimental Morphology (JEEM). JEEM was launched in 1953 and renamed Development in 1987. Two articles to be published in Development later in the year will provide a more detailed history of the journal, as well as a deeper look into the decision behind changing the journal's name.

At this point in the early 1950s, the Company owned three journals printed by two different publishers: JEB, published by Cambridge University Press; and QJMS and JEEM, published by Clarendon Press in Oxford. In 1963, publication of JEEM was transferred to Cambridge University Press, with JCS following in 1966. Roughly a decade later, the Company began to discuss printing its own journals and made the bold move to set up its own printing house in 1983 within a terraced house in the centre of Cambridge. In an early example of engaging early-career researchers, the Company often paid graduate studies to staple reprints, occasionally initiating long-term associations with the Company.

**Fig. 2.**
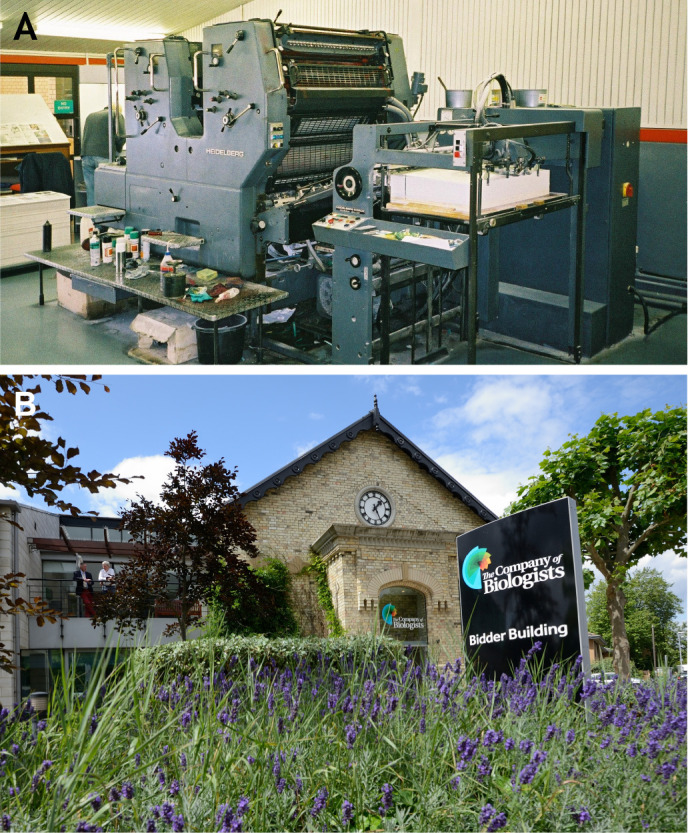
**The Editorial offices of The Company of Biologists.** (A) The Company of Biologists’ printing press at Cowley Road in Cambridge, UK. Photo taken by Richard Sever during his employment at the Company. (B) The Company of Biologists' current offices in Histon, just outside Cambridge, UK.

The press moved to a more appropriate location on the outskirts of Cambridge in the mid-1980s, which would house its own printing press a few years later (Fig. 2A). Technology continued to develop at a record pace, with the Company producing its first electronic issues (on CD-ROM) alongside print copies in 1992, launching its first website and online issues in 1997, and adopting an online journal-hosting platform and manuscript submission system in 2001 and 2006, respectively. The journals are now online-only and the entire archive from all our journals has been digitised and indexed, and is fully searchable with content dating back to 1853 (QJMS).

In the new millennium, conversations regarding the increasing popularity of developing organisms to model human disease highlighted the need for a scientific venue for such research (Freeman and St Johnston, 2008). These discussions culminated in 2008 with the release of a new journal, Disease Models & Mechanisms (DMM), which transitioned to being the Company's first Gold Open Access (OA) journal in 2011 (Siegel, 2011). In later issues of DMM, we will follow the journal's journey from its founding to its current position in publishing cutting-edge research in disease biology, along with DMM's community-driven efforts at supporting early-career researchers.

In 2012, the Company launched its first online-only and born-OA journal, Biology Open (BiO), to combat the problem of unpublished data going to waste by accepting good-quality research spanning the whole field of biology – independent of perceived impact or novelty (Raff, 2012). In the next issue of BiO, the journal's past and present Editors-in-Chief will reflect on key milestones and share personal insights on the major successes and challenges during their tenures.

**Fig. 3.**
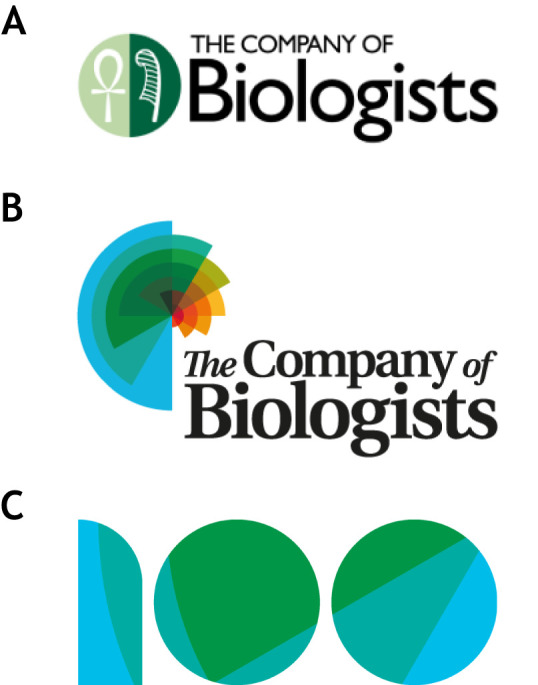
**The Company of Biologists' identity through time.** (A) The Company of Biologists' logo before 2015 features the Company seal (see Fig. 1A). (B) The current logo of The Company of Biologists features the colourful ‘nautilus’ symbol. (C) A special logo for The Company of Biologists commemorating its 100-year anniversary.

Although each of our journals has come to be known and recognised in their communities, for a long time, The Company of Biologists was largely unfamiliar to authors. A decade ago, concurrent with the move to a professional editorial office in Histon, just outside Cambridge (Fig. 2B), a project was launched to unite The Company of Biologists and its journals under a single umbrella – the recognisable brand you know today. Retiring the Company seal as a logo (Fig. 3A), the characteristic and colourful ‘nautilus’ (Fig. 3B) was introduced. You may recognise elements of this design in the ‘100’ logo created in celebration of the 100-year anniversary (Fig. 3C).

## Supporting biologists: the Company at 100

Continuing to follow the same ethos of ‘by scientists, for scientists’, today, the Company is steered by a group of expert practising scientists who form the Board of Directors (https://www.biologists.com/about-us/). When advertising for new Board members, applications usually come from members of the community who have interacted with our journals and are interested in supporting us as a not-for-profit publisher and UK charity. Directors contribute their time without payment and, in addition to quarterly meetings of the full Board, are involved in sub-committees such as the Grants Committee or Workshops Committee (discussed below), as well as supporting the in-house staff and the journals' Editors-in-Chief.

Although still a relatively modestly sized publisher with about 70 employees, The Company of Biologists has successfully navigated the ever-changing publishing landscape to achieve international reach and recognition for its journals. Indeed, in 2009, Development and JEB were each voted one of the most influential biomedical journals in the past century (Knight, 2009) and, more recently, Development, JCS and JEB were the first journals to be awarded Transformative Journal status by Plan S, pioneering new Read & Publish agreements that provide authors fee-free OA publication options (Moulton and Brown, 2020; Moulton and Ahmad, 2020; Moulton and Handel, 2020). The Company has continued to embrace changes in the publication ecosystem, strongly supporting the preprint revolution in the life sciences by developing preprint-friendly journal policies and launching the preprint-highlighting service preLights in 2018 – shortlisted for an Innovation in Publishing Award from the Association of Learned and Professional Society Publishers (ALPSP).

As a not-for-profit publisher, the Company is recognised not only for its leading journals, but also for a wide range of charitable activities, such as Travelling Fellowships and meeting grants, as well as for organising an annual programme of Workshops and Journal Meetings with a charitable budget of over £3 million this year (Box 2). In addition to preLights, we host two other community sites to foster communication, collaboration and community building: the Node – a community site for developmental and stem cell biologists – launched in 2010 (Amsen et al., 2010); and, most recently, FocalPlane, launched in 2020 for biological microscopy and image analysis (Ahmad et al., 2020). Finally, the Company has continued to listen to the concerns and motivations of the community, resulting in fostering exciting innovations, such as the Sustainability Initiative and The Forest of Biologists (Moulton and Freeman, 2023), a biopositive publishing project that has been ‘highly commended’ by the 2024 ALPSP Impact Award. You can learn more about how and why these charitable and community activities have been introduced, as well as their impact on the research and researchers, later in 2025.

**Box 2. Charitable and community activities from The Company of Biologists**

The 2025 charitable budget from The Company of Biologists exceeds £3 million. How is this money distributed?

**Society grants**

The Company of Biologists funds various societies, both large and small. Three of the societies we fund (the British Society for Developmental Biology, the British Society for Cell Biology and the Society for Experimental Biology) use part of our funding to provide travel grants to support early-career scientists who wish to attend conferences.

**Charitable grants**

•Scientific Meeting Grants: https://www.biologists.com/grants/scientific-meeting-grants/

•Small Meeting Grants: https://www.biologists.com/grants/small-meeting-grants/

• Travelling Fellowships: https://www.biologists.com/grants/travelling-fellowships/

• DMM Conference Travel Grants: https://www.biologists.com/grants/dmm-conference-travel-grants/

• JCS-FocalPlane Training Grants: https://www.biologists.com/grants/jcs-focalplane-training-grants/

• JEB Grants for junior faculty staff, which include:

  o  Research Partnership Kickstart Travel Grants: https://www.biologists.com/grants/kickstart-travel-grants/

  o  ECR Visiting Fellowships: https://www.biologists.com/grants/ecr-visiting-fellowships/

• Development's Pathway to Independence Programme: https://www.biologists.com/grants/development-pathway-independence/

**Community building**

• The Node: https://thenode.biologists.com/

• preLights: https://prelights.biologists.com/

• FocalPlane: https://focalplane.biologists.com/

• Workshops programme: https://www.biologists.com/workshops/

• Journal Meetings: https://www.biologists.com/meetings/

**Sustainability and biodiversity**

• The Forest of Biologists: https://forest.biologists.com/

• The Company of Biologists' Sustainability Initiative: https://www.biologists.com/sustainability-hub/

• The Company of Biologists' Sustainability Grants: https://www.biologists.com/sustainability-hub/sustainability-initiative/grants/

A landmark of our 100th birthday year will be our ‘Biologists @ 100’ conference. This, the largest ever of the Company's meetings, brings together the communities served by each of the five journals. It encompasses the Spring Meetings from the BSCB and BSDB, a JEB Symposium – ‘Sensory perception in a changing world’, and a 1-day disease-themed programme – ‘Interdisciplinary approaches to combatting antimicrobial resistance’, as well as a SEB satellite symposium – ‘Experimental biology and impact: solutions to climate change and biodiversity loss’. Registration is open until 28 February 2025 – we hope that you will join the celebrations by registering at https://100yearsconference.biologists.com/.

## Inspiring biology: the next 100 years

What does the future hold in store for The Company of Biologists? It is easy to celebrate our history and all the extraordinary science, ideas and people that have brought us to where we are now – it is much harder to project ahead for the next 10 or 20 years, let alone the next 100 years. With the shifting sands in scientific publication and the technological landscape, we must remain nimble-footed. Adapting to change will continue to be a constant as we adapt to the challenges posed by the OA movement, further integrate preprinting into the life cycle of an article, and experiment with the opportunities provided by advances in artificial intelligence (AI). Communities will always sit at the core of what we do – from the smaller groups we bring together at Workshops, to the authors and readers of each of our journals and to the wider global scientific community beyond. Of particular importance in the current world is growing sustainable practices and promoting better geographical equality, diversity and inclusion in our activities (Lowell et al., 2022). The recent launch of a Global South Workshops programme (Bars-Closel et al., 2024), Article Publication Charge (APC) waivers in our OA journals, free ‘Green’ OA publication in our transformative journals, and Read & Publish agreements with Electronic Information for Libraries (EIFL) that provide fee-free Gold OA publication in all of our journals for many developing and transition economy countries are steps in that direction.

Our communities remain key to the success of The Company of Biologists; you help us define our future. As authors and readers, you can support and shape our journals by (1) submitting your manuscripts, (2) accepting invitations to peer review and write, and (3) reading and citing our content. We also encourage you to take advantage of the charitable grants we have on offer (Box 2) and, as delegates at our scientific meetings, to contribute to the important scientific discussion, networking and discovery that feeds the biological community. One thing we do know is that supporting biologists and inspiring biology will remain at the heart of The Company of Biologists for years to come.

## Supplementary Material

10.1242/dmm.052228_sup1 Poster
