# Supplementary figures and images for: The Company of Biologists: celebrating 100 years
Source: Dis Model Mech. 2025 Jan 6;18(1):dmm052228. doi: 10.1242/dmm.052228 (PMC11804787; doi:10.1242/dmm.052228)

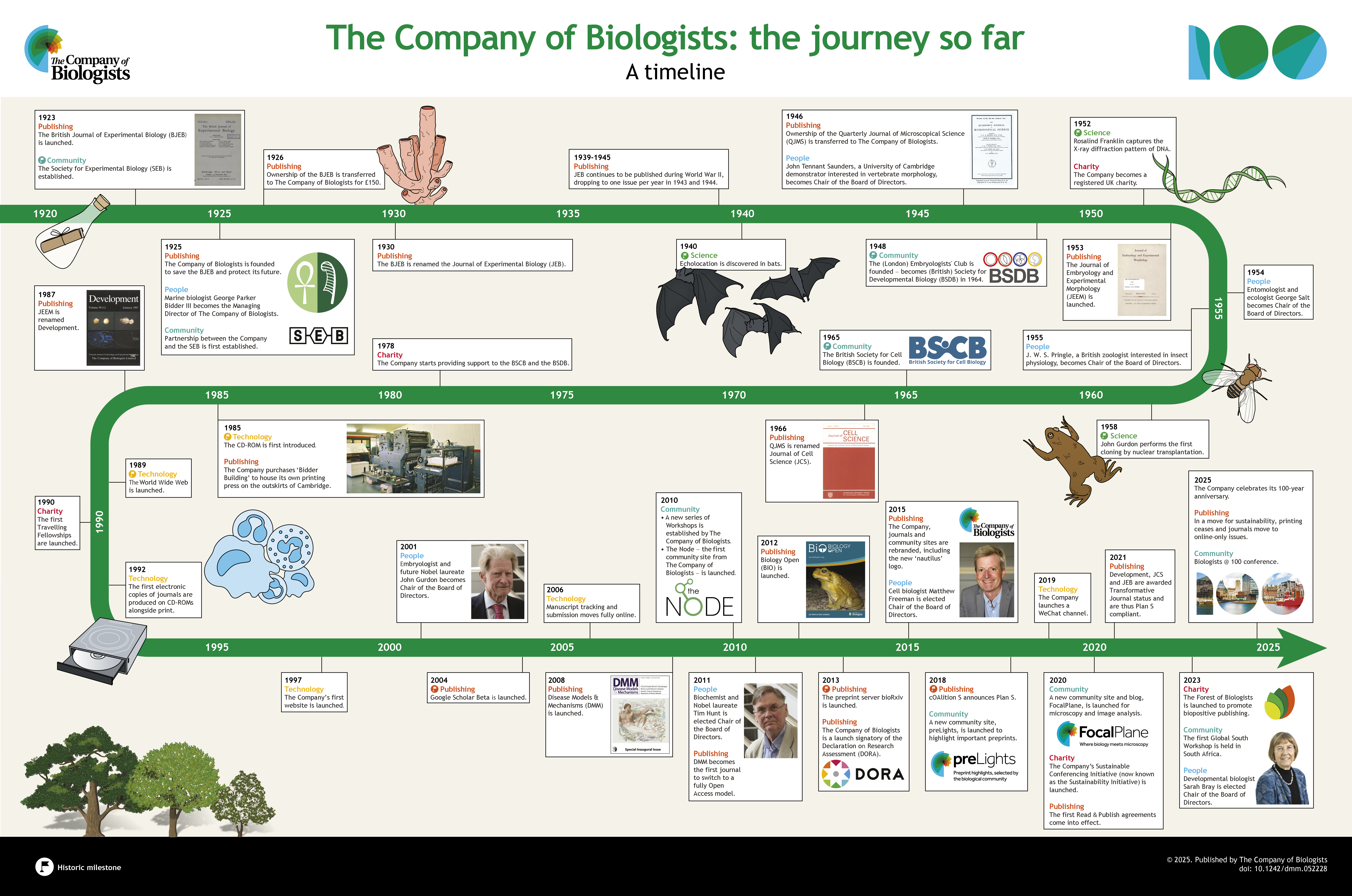

Supplement: Poster [file dmm-18-052228-s1.jpg]
